# Transcriptome analysis identification of A-to-I RNA editing in granulosa cells associated with PCOS

**DOI:** 10.3389/fendo.2023.1170957

**Published:** 2023-07-21

**Authors:** Fan-Sheng Kong, Zijing Lu, Yuan Zhou, Yinghua Lu, Chun-Yan Ren, Ruofan Jia, Beilei Zeng, Panwang Huang, Jihong Wang, Yaping Ma, Jian-Huan Chen

**Affiliations:** ^1^ Department of Pediatrics, Affiliated Hospital of Jiangnan University, Wuxi, Jiangsu, China; ^2^ Laboratory of Genomic and Precision Medicine, Wuxi School of Medicine, Jiangnan University, Wuxi, Jiangsu, China; ^3^ Department of Ophthalmology, Affiliated Hospital of Jiangnan University, Wuxi, China; ^4^ Department of Reproductive Medicine, Affiliated Hospital of Jiangnan University, Wuxi, Jiangsu, China; ^5^ Joint Primate Research Center for Chronic Diseases, Institute of Zoology of Guangdong Academy of Science, Jiangnan University, Wuxi, Jiangsu, China; ^6^ Jiangnan University Brain Institute, Wuxi, Jiangsu, China

**Keywords:** PCOS, epigenetics, *cis*-regulatory analysis, A-to-I RNA editing, apoptosis

## Abstract

**Background:**

Polycystic ovary syndrome (PCOS) is a complex, multifactor disorder in women of reproductive age worldwide. Although RNA editing may contribute to a variety of diseases, its role in PCOS remains unclear.

**Methods:**

A discovery RNA-Seq dataset was obtained from the NCBI Gene Expression Omnibus database of granulosa cells from women with PCOS and women without PCOS (controls). A validation RNA-Seq dataset downloaded from the European Nucleotide Archive Databank was used to validate differential editing. Transcriptome-wide investigation was conducted to analyze adenosine-to-inosine (A-to-I) RNA editing in PCOS and control samples.

**Results:**

A total of 17,395 high-confidence A-to-I RNA editing sites were identified in 3,644 genes in all GC samples. As for differential RNA editing, there were 545 differential RNA editing (DRE) sites in 259 genes with Nucleoporin 43 (*NUP43*), Retinoblastoma Binding Protein 4 (*RBBP4*), and leckstrin homology-like domain family A member 1 (*PHLDA*) showing the most significant three 3′-untranslated region (3′UTR) editing. Furthermore, we identified 20 DRE sites that demonstrated a significant correlation between editing levels and gene expression levels. Notably, MIR193b-365a Host Gene (*MIR193BHG*) and Hook Microtubule Tethering Protein 3 (*HOOK3*) exhibited significant differential expression between PCOS and controls. Functional enrichment analysis showed that these 259 differentially edited genes were mainly related to apoptosis and necroptosis pathways. RNA binding protein (RBP) analysis revealed that RNA Binding Motif Protein 45 (RBM45) was predicted as the most frequent RBP binding with RNA editing sites. Additionally, we observed a correlation between editing levels of differential editing sites and the expression level of the RNA editing enzyme Adenosine Deaminase RNA Specific B1 (*ADARB1*). Moreover, the existence of 55 common differentially edited genes and nine differential editing sites were confirmed in the validation dataset.

**Conclusion:**

Our current study highlighted the potential role of RNA editing in the pathophysiology of PCOS as an epigenetic process. These findings could provide valuable insights into the development of more targeted and effective treatment options for PCOS.

## Introduction

RNA editing is an epigenetic alteration of the RNA nucleotide sequence with nucleotide insertions, deletions, or substitutions (1). In mammals, canonical RNA editing includes adenosine-to-inosine (A-to-I) editing and cytidine-to-uridine (C-to-U) editing (2). Altered A-to-I editing has been implicated in various diseases, including autoimmune disorders, cardiovascular diseases, neurological diseases, and cancers, suggesting its involvement in the molecular mechanisms of these pathological processes (3–6).

Polycystic ovary syndrome (PCOS) is a complex multigenic disorder characterized by excessive androgen levels and ovarian dysfunction (7). It is the most common endocrine-metabolic disorder in women of reproductive age in the world (8). Granulosa cells (GCs) play a crucial role in the pathogenesis of PCOS. Studies have demonstrated that atrial natriuretic peptides can inhibit GC apoptosis to modulate ovarian function in PCOS (9). Furthermore, emerging studies show epigenetic modifications and altered gene expression patterns in GCs from PCOS women and mouse models, suggesting a potential contribution of epigenetic mechanisms to PCOS development (10, 11). However, understanding A-to-I RNA editing in the context of PCOS remains limited. Further investigation is needed to elucidate the role of A-to-I RNA editing in PCOS development and progression.

To identify A-to-I RNA editing associated with PCOS, we performed a transcriptome-wide analysis of RNA-Seq data from ovarian GCs and validated the findings using a cross-cohort approach. Our findings revealed dramatic A-to-I RNA editing alterations in PCOS compared to controls and underlined their substantial role in the epigenetic regulation of PCOS.

## Materials and methods

### Data collection

We searched the OmicsDI database and found two RNA-Seq datasets of GCs from PCOS and control women. The dataset GSE138518 retrieved from the NCBI Gene Expression Omnibus (GEO, http://www.ncbi.nlm.nih.gov/geo) database was used for RNA editing event discovery and consisted of GCs from adult women, including five with PCOS and six controls without PCOS undergoing *in vitro* fertilization treatments (IVF) (12). In addition, the validation dataset PRJNA762274 was retrieved from the European Nucleotide Archive Databank (https://www.ebi.ac.uk/), which contained GCs from four PCOS patients and four controls undergoing IVF or intracytoplasmic sperm injection (13). The processes of RNA extraction, library construction, and RNA sequencing were described in the original studies.

### RNA-Seq data processing

After the raw sequencing data were retrieved, FASTQC was first used to analyze the raw data for quality control. Adaptor and low-quality sequences were removed using FASTP Version 0.23.4 (14). RNA STAR (Version 2.7.0e) was used to map sequencing reads to the human reference genome (UCSC hg38) and generate alignment files in Binary Alignment Map (BAM) format (15). The BAM files were processed using SamTools (Version 1.9) to remove optic duplications and retain only reads uniquely mapped to the human reference genome (16). GATK (Version 4.1.3) was used to recalibrate the base quality scores of the BAM files by following the instructions provided in the GATK best practice guidance (17).

### Identification of high-confidence A-to-I RNA editing events

Variant calling was then conducted to identify candidates for RNA editing events. Single-nucleotide variation (SNV) was called from the BAM files by using VarScan (Version 2.4.3) as described in our previous study (18). The variant calling criteria were defined as follows: a minimum base quality of 25, a total sequencing depth of at least 10, an alternative allele depth of 2 or more, and an alternative allele frequency (AAF) of 1% or higher. To eliminate potential false-positive SNVs, VarScan was employed with its default parameters to filter and remove them. The SNVs were then annotated using the Ensembl Variant Effect Predictor (VEP) (19). SNVs were further filtered and removed according to the criteria as follows unless annotated as RNA editing sites in the REDIportal V2.0 database: (1) located in homopolymer runs ≥ five nucleotides (nt), simple repeats, or the mitochondria, (2) within six nt from splice junctions, one nt from insertions or deletions, or 4% to the ends of reads; (3) annotated in the dbSNP database Build 142; (4) more than 90% of the samples had an AAF equal to 100% or between 40% and 60% (20–22). The remaining high-confidence A-to-I (recognized as A-to-G transition in the RNA-Seq reads) RNA editing events were kept if the editing levels were observed ≥ 1% in at least two samples (20, 21).

### Gene expression quantification

To quantify the gene expression levels, FeatureCounts Version 2.0.1 was used to calculate the pseudo-counts of gene expression from the BAM files, and EdgeR (Version 3.7) was then used to calculate the value of transcripts read per thousand bases per million mappings (TPM) for each gene (23, 24).

### Principal component analysis

To evaluate how RNA editing could contribute to the difference between PCOS and controls, the principal component analysis (PCA) of A-to-I RNA editing events was performed using the function prcomp of R (Version 3.6.3) and visualized using the ggplot2 (Version 2.2.1) package.

### Gene function enrichment analysis

To understand the possible functional relevance of RNA editing, gene ontology (GO) and Kyoto Encyclopedia of Genes and Genomes (KEGG) pathways of edited genes were analyzed using Enrichr (25). Items with enrichment *p* < 0.05 were considered significant and visualized using the online tools provided at the website (http://www.bioinformatics.com.cn/).

### Prediction of RNA secondary structures and RNA binding protein binding

To evaluate the potential functional impact of RNA editing, the RNAfold web server (http://rna.tbi.univie.ac.at/cgi-bin/RNAWebSuite/RNAfold.cgi) was used to predict secondary structures of single-stranded RNA sequences surrounding RNA editing sites (26, 27). To further understand the potential functional impact of RNA editing, RBPmap (http://rbpmap.technion.ac.il) was used to predict the binding of RNA binding protein (RBP) to differential RNA editing sites (28). The results of RBP binding prediction were visualized using the wordcloud package (Version 2.6).

### Statistical analysis

To identify differential RNA editing, the intergroup RNA editing levels were compared using the generalized linear model (GLM), and the likelihood ratio test (LRT) was used. The *t*-test was used to compare the gene expression intergroup levels. *p* < 0.05 was used as the significance cutoff. The correlation between RNA editing and gene expression was analyzed using the Spearman’s correlation method to calculate the correlation coefficient (*r*) and *p*-value.

## Results

### Identification of A-to-I RNA editing events in GCs

**Figure 1:**
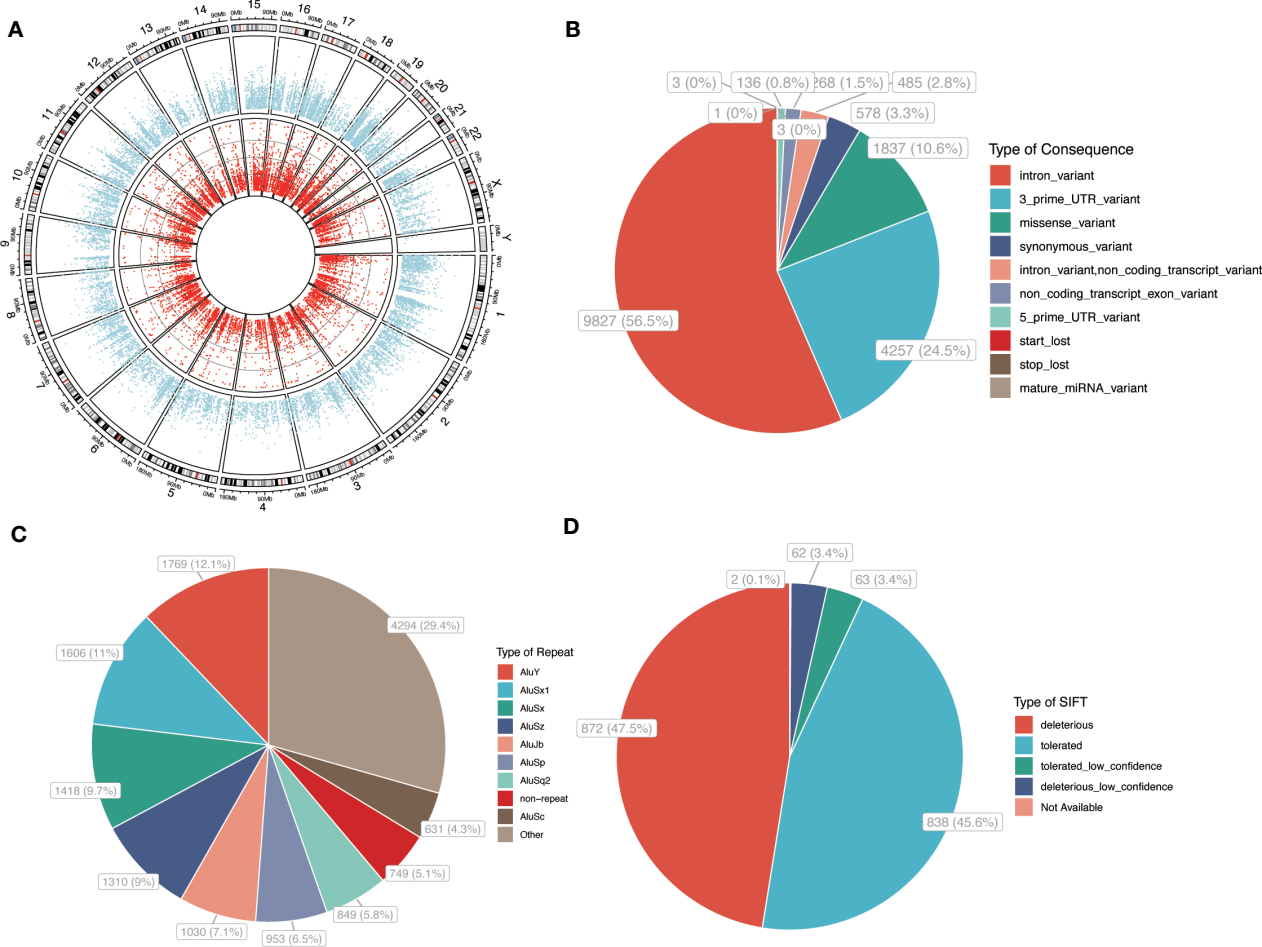
A-to-I RNA editing events in GC. **(A)** Circos plot of gene expression and A-to-I RNA editing sites in PCOS and controls. Outer circle: average level of gene expression. Inner circle: A-to-I RNA editing sites. **(B)** A-to-I RNA editing functional categories. **(C)** repetitive elements overlapped with RNA editing sites. **(D)** SIFT prediction of that missense variants. P, PCOS; C, controls.

Our analysis identified 17,395 high-confidence A-to-I RNA editing events in 3,644 genes in GCs (**Figure 1A**). The SNV density across various chromosomes is shown in **Supplementary Figure S1**. Regarding the genomic distribution of the identified editing sites, the majority (56.5%) were annotated as intronic variants, while 24.5% were located in the 3′-untranslated regions (3′UTR). The remaining variants were distributed across other regions (**Figure 1B**). Notably, 65.5% of all editing sites were located in Alu repetitive elements (**Figure 1C**). Sorting intolerant from tolerant (SIFT) algorithm was utilized to predict the functional impact of missense variants, revealing that 50.9% of the missense variants were predicted to be deleterious (including both deleterious and low confidence deleterious variants) and might potentially affect the encoded protein (**Figure 1D**; **Supplementary Table S1**).

We then conducted a motif analysis of the sequence between 6 bp upstream and downstream of the editing sites. Our findings indicated that in most categories, G was suppressed 1 bp upstream of the editing sites (**Supplementary Figure S2**).

### Comparison between PCOS and control GCs identified PCOS-associated A-to-I RNA editing

**Figure 2:**
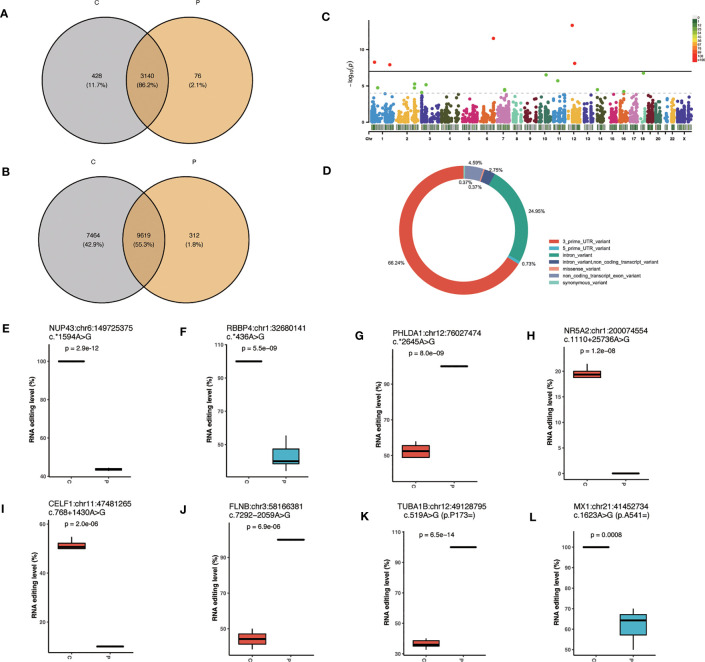
Differential A-to-I RNA editing sites in PCOS and controls. **(A, B)** Venn plots of A-to-I RNA editing events in PCOS and controls. **(C)** Manhattan plot of differential RNA editing sites across the chromosomes. **(D)** Types of mRNA variants resulted from 545 differential A-to-I RNA editing events. **(E–G)** boxplots of the top 3 3′UTR editing sites (NUP43:chr6:149725375, RBBP4:chr1:32680141, and PHLDA1:chr12:76027474). **(H–J)** Boxplots of the top three intronic editing sites (NR5A2:chr1:200074554, CELF1:chr11:47481265, and FLNB:chr3:58166381). **(K, L)** Boxplots of synonymous editing sites (TUBA1B:chr12:49128795 and MX1:chr21:41452734). Blue and red represent the groups of PCOS and control, respectively. P, PCOS; C, controls.

By comparing the RNA editing between PCOS and control GCs, a lower number of editing sites and genes unique to PCOS was observed. In total, 428 genes were uniquely edited in controls, whereas 76 genes were uniquely edited in PCOS. Likewise, 7,464 editing sites were unique to controls, whereas 312 were unique to PCOS (**Figures 2A, B**).

In the GLM and LRT results, 545 sites in 259 genes exhibited differential A-to-I RNA editing between PCOS and control GCs (**Figure 2C**; **Supplementary Table S2**). These differential RNA editing (DRE) sites included various consequence types, with the majority (66.24%) located in the 3′UTR and a significant portion (24.95%) in intronic regions (**Figure 2D**). The most significant 3′UTR variants were found in Nucleoporin 43 (*NUP43*), Retinoblastoma Binding Protein 4 (*RBBP4*), and leckstrin homology-like domain family A member 1 (*PHLDA*), including *NUP43*:chr6:149725375, *RBBP4*:chr1:32680141, and *PHLDA1*:chr12:76027474 (**Figures 2E–G**). The top three intronic variants were found in Nuclear Receptor Subfamily 5 Group A Member 2 (*NR5A2*), CUGBP Elav-Like Family Member 1 (*CELF*), and Filamin B, (*FLNB*), including *NR5A2*:chr1:200074554, *CELF1*:chr11:47481265, and *FLNB*:chr3:58166381 (**Figures 2H–J**). In addition, we also identified two synonymous variant sites in Tubulin Alpha 1b (*TUBA1B*), and MX Dynamin Like GTPase 1 (*MX1*), including *TUBA1B*:chr12:49128795 and *MX1*:chr21:41452734 (**Figures 2K, L**).

Additionally, we performed PCA using these differential RNA editing sites. The results showed that PC1 and PC2 accounted for 73.28% and 13.03% of the total variance, respectively (**Supplementary Figure S3**).

### Correlation analysis between PCOS-associated RNA editing and gene expression levels

**Figure 3:**
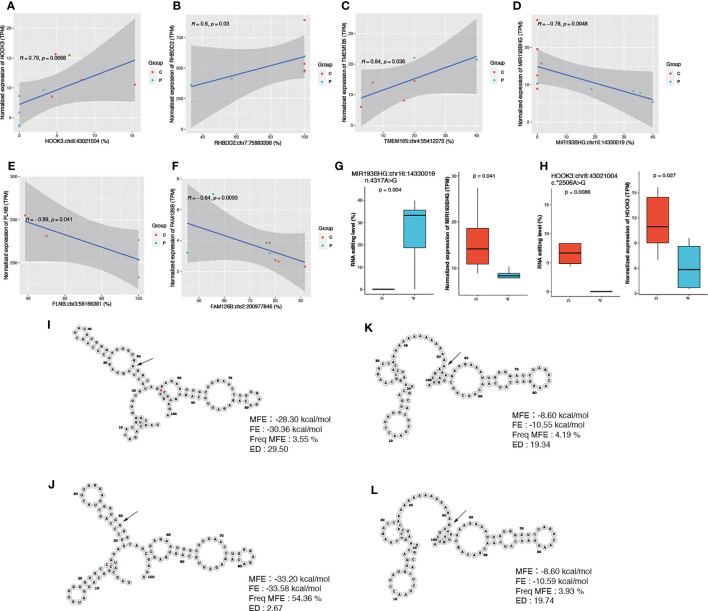
Differential editing sites with cis-regulatory effects on gene expression. **(A–F)** Scatter plots showing their RNA editing and gene expression levels in PCOS and controls. **(G, H)** The RNA editing and expression levels of MIR193BHG and HOOK3. **(I, J)** The RNA structure and stability parameters before and after RNA editing for HOOK3:chr8:43021004 and **(K, L)** MIR193BHG: chr16:14330019. P, PCOS; C, controls; MFE, minimum free energy; FE, free energy of the thermodynamic ensemble; Freq MFE, frequency of the MFE structure in the ensemble; ED, ensemble diversity.

We then conducted the correlation to explore the relationship between RNA editing and gene expression levels. Our findings revealed significant correlations between editing sites and gene expression levels for 20 DRE sites (*p* < 0.05). Among these sites, eight showed a positive correlation, while the remaining 12 exhibited a negative correlation. Noteworthy, the top three editing sites with positive correlations were found in Hook Microtubule Tethering Protein 3 (*HOOK3*), Rhomboid Domain Containing 2 (*RHBDD2*), and Transmembrane Protein 16 (*TMEM165*), including *HOOK3*:chr8:43021004 (*r* = 0.79), *RHBDD2*:chr7:75883398 (*r* = 0.8), and *TMEM165*:chr4:55412273 (*r* = 0.84) (**Figures 3A–C**).

On the other hand, sites in *FLNB*, Hyccin PI4KA Lipid Kinase Complex Subunit 2 (HYCC2, also called *FAM126B*), and *MIR193BHG*, including, *FLNB*:chr3:58166381 (*r* = −0.89), *FAM126B*:chr2:200977846 (*r* = −0.84), and *MIR193BHG*:chr16:14330019 (*r* = −0.78) showed the top three significant negative correlations with gene expression (**Figures 3D–F**). The editing levels of these 20 sites are shown in **Supplementary Figure S4**.

Furthermore, we examined the expression levels of genes containing the identified editing sites and observed significantly lower expression levels of *MIR193BHG* and *HOOK3* in PCOS compared to those in controls (**Figures 3G, H**). We then performed prediction analysis to investigate whether RNA editing changed the stability of the mRNA secondary structures of *HOOK3* and *MIR193BHG* before and after editing. The detailed information of the two editing sites (*HOOK3:chr8:43021004* and *MIR193BHG:chr16:14330019*) including the minimum free energy (MFE), the free energy of the thermodynamic ensemble (FE), the frequency of the MFE structure in the ensemble, and ensemble diversity is presented in **Figures 3I–L**.

### Correlation analysis between RNA editing enzyme expression and PCOS-associated RNA editing

**Figure 4:**
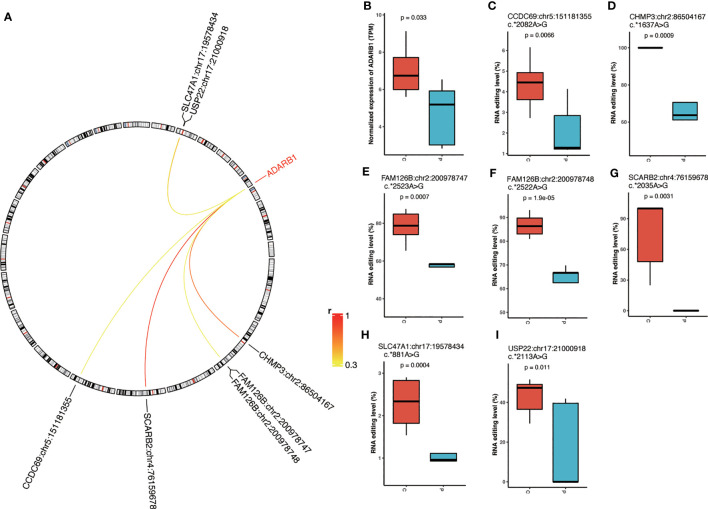
The correlation between RNA editing enzyme expression and differential editing sites. **(A)** The correlation between *ADARB1* expression and differential A-to-I RNA editing sites. **(B)** The expression level of *ADARB1*. **(C–I)** The editing level of *ADARB1*-related DRE sites. P, PCOS; C, controls.

We further assessed the expression of editing enzymes, including Adenosine Deaminase RNA Specific (*ADAR*) and Adenosine Deaminase RNA Specific B1 (*ADARB1*). We observed a significant decrease in *ADARB1* expression in PCOS compared to controls (**Figure 4A**). Therefore, we further looked into the correlation between *ADARB1* expression and PCOS-associated RNA editing and identified six ADARB1-related sites in Scavenger Receptor Class B Member 2 (*SCARB2*), Charged Multivesicular Body Protein 3 (*CHMP3*), Ubiquitin Specific Peptidase 22 (*USP22*), *FAM126B*, Solute Carrier Family 47 Member 1 (*SLC47A1*), and Coiled-Coil Domain Containing 69 (*CCDC69*), including *SCARB2*:chr4:76159678, *CHMP3*:chr2:86504167, *USP22*:chr17:21000918, *FAM126B*:chr2:200978748, *FAM126B*:chr2:200978747, *SLC47A1*:chr17:19578434, and *CCDC69*:chr5:151181355, all with significantly downregulated RNA editing correlated to the decreased *ADARB1* expression (**Figures 4B–I**).

### Functional enrichment of PCOS-associated RNA editing

**Figure 5:**
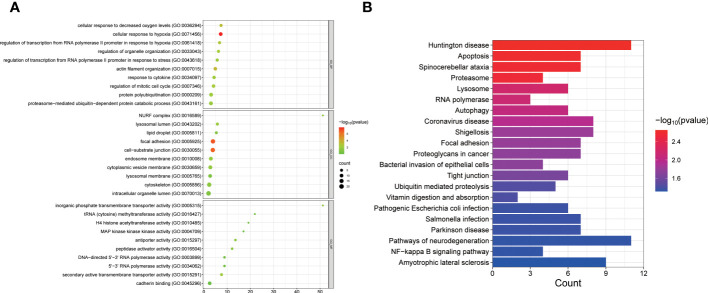
GO **(A)** and KEGG pathways **(B)** enriched by genes with differential A-to-I RNA editing between PCOS and controls.

We performed enrichment analysis to gain insights into the biological functions of PCOS-associated A-to-I RNA editing in GCs. Our results revealed that the differentially edited genes were primarily enriched in biological processes related to the regulation of the mitotic cell cycle and transmembrane transporter processes (**Figure 5A**). Moreover, the most significantly enriched KEGG pathways of the differentially edited genes included apoptosis, necroptosis, and coronavirus disease (**Figure 5B**), implicating a potential link between PCOS and coronavirus disease.

### RBP binding prediction of differential editing sites

**Figure 6:**
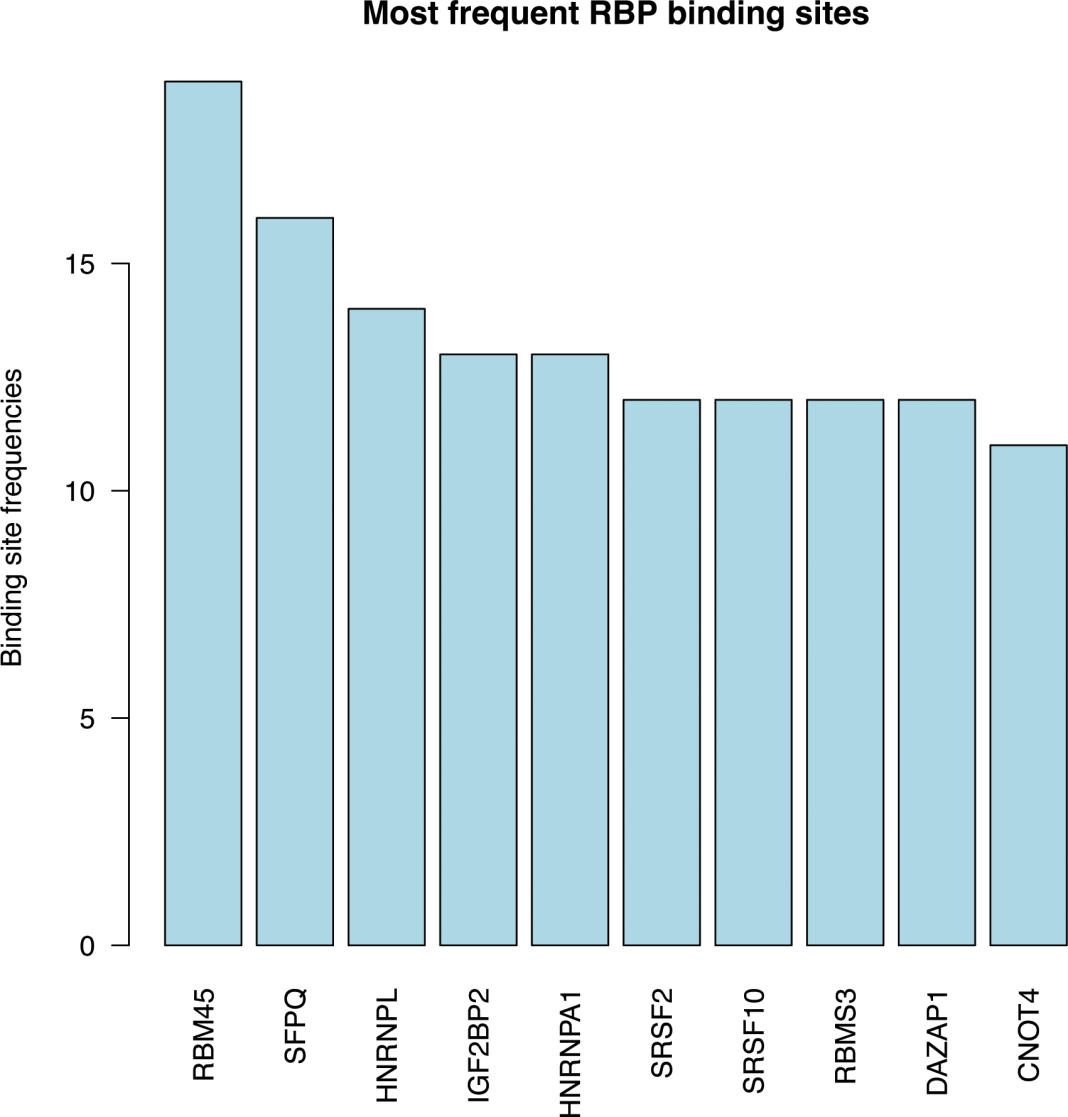
RNA-binding protein (RBPs) prediction showing the top 10 frequent RBPs binding to DRE sites.

To gain insights into the potential mechanism of PCOS-associated RNA editing, we employed the RBPsmap website to predict the RBP binding of the identified RNA editing sites (**Supplementary Figure S5**). **Figure 6** illustrates the top 10 RBPs with the highest binding frequency of differential RNA editing sites. Notably, RNA binding motif protein 45 (RBM45), splicing factor proline and glutamine rich (SFPQ), and heterogeneous nuclear ribonucleoprotein L (HNRNPL) were found to most frequently bind to the PCOS-associated RNA editing sites.

### Validation of PCOS-associated RNA editing

**Figure 7:**
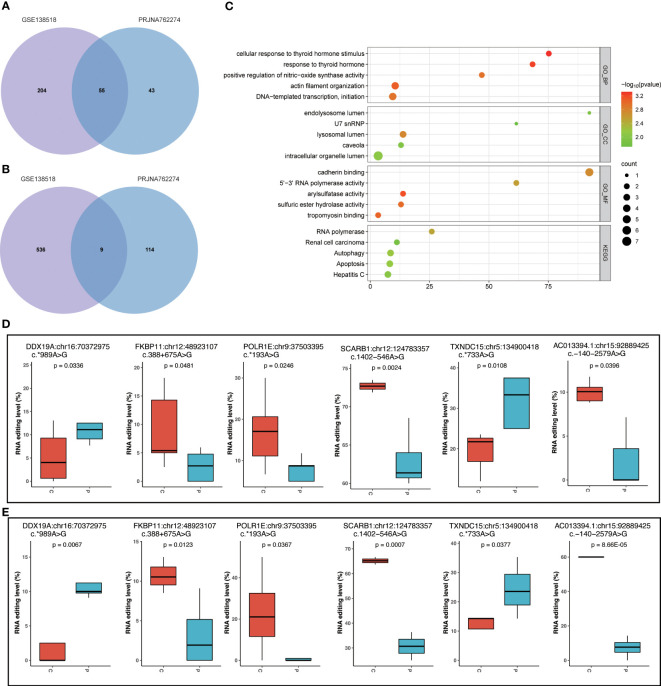
Validation of RNA editing events in dataset PRJNA762274. **(A)** Venn plot of differential edited genes between datasets GSE138518 and PRJNA762274. **(B)** Venn plot of differential editing sites between datasets GSE138518 and PRJNA762274. **(C)** The top 5 functional analysis terms of KEGG and GO for the 55 common differentially edited genes shared by the two datasets. **(D)** Six common differential RNA editing sites with the same differential editing trend in dataset GSE138518. **(E)** Six common differential RNA editing sites with the same differential editing trend in dataset PRJNA762274. P, PCOS; C, controls.

To validate the common characteristics of RNA editing, we conducted a comparative analysis using another PCOS and control GC sample dataset (PRJNA762274) from an independent study (13). By using the same analysis procedure, we identified the differential editing sites shown in **Supplementary Table S3**. To assess their functional relevance, we then performed enrichment analysis on the common set of differentially edited genes shared between the discovery dataset GSE138518 and the validation dataset PRJNA762274. The Venn plots (**Figures 7A, B**) show the overlap between the discovery and validation datasets, revealing 55 differentially edited genes and nine differential editing sites shared by both datasets. Furthermore, the enrichment analysis found that these 55 edited genes were mainly involved in apoptosis, the HIF-1 signaling pathway, cellular response to thyroid hormone stimulus, and arylsulfatase activity (**Figure 7C**). Among the nine differential editing sites, six in Scavenger Receptor Class B Member 1 (*SCARB1*), Thioredoxin Domain Containing 15 (*TXNDC15*), RNA Polymerase I Subunit E (POLR1E), DEAD-Box Helicase 19A (*DDX19A*), and FKBP Prolyl Isomerase 11 (*FKBP11*) showed consistent changes between the two datasets, namely *SCARB1*:chr12:124783357, *TXNDC15*:chr5:134900418, *POLR1E*:chr9:37503395, *DDX19A*:chr16:70372975, *AC013394.1*:chr15:92889425, and *FKBP11*:chr12:48923107 (**Figures 7D, E**).

## Discussion

Understanding the molecular mechanism of PCOS could help develop new stratiges for diagnosis and treatment of PCOS (29, 30). Several biomarkers associated with PCOS have been identified and might serve as potential therapeutic targets. RNA editing has been reported to play a role in cancer, aging, neurological, and autoimmune diseases, yet how it is involved in PCOS remains poorly understood (31). In this study, we systematically investigated and validated A-to-I RNA editing associated with PCOS as biomarkers at the transcriptome level, providing valuable insights into the underlying mechanisms of PCOS.

RNA editing dysfunction, particularly A-to-I RNA editing events, has been implicated in various diseases, including immune signaling pathways and certain cancers (32–35). Recent studies have shown that synonymous variants can also affect mRNA splicing, mRNA stability, and protein function (36–38). We identified two synonymous variants, *TUBA1B*:chr12:49128795 and *MX1*:chr21:41452734, while the role of the two in PCOS is unclear. However, *TUBA1B*, a critical gene in postmenopausal osteoporosis, has also been linked to BMI in children’s muscles and the overall survival of colorectal cancer patients (39–41). The expression level of *MX1* in cumulus-oophorus cells was related to the immunological defense processes (42). Regarding the 3′UTR RNA editing, *NUP43*:chr6:149725375, *PHLDA1*:chr12:76027474, and *RBBP4*:chr1:32680141 were the top three significant editing sites. *PHLDA1* has been reported to possibly contribute to PCOS phenotypes and regulate proinflammatory cytokine production by interacting with Tollip (43, 44). *RBBP4* is involved in apoptosis in early mouse embryonic development, and *NUP43* plays a crucial role in various cancers, including breast cancer and gastric cancer (45–47). Further study is necessary to determine the roles of these genes and their RNA editing in PCOS.

Our *cis*-Regulation analysis showed that 20 DRE sites were significantly related to the gene expression level. Notably, the expression levels of *MIR193BHG* and *HOOK3* showed significant differences between the two groups. Previous studies have identified *MIR193BHG* as a prognostic marker in pancreatic, ovarian, and head and neck squamous cell carcinoma (48–50). Additionally, *MIR193BHG* might play a pivotal role in preeclampsia related to blood pressure and urine protein and could be a potential prognostic biomarker in early-onset preeclampsia (51, 52). The phosphorylation of *HOOK3* has been shown to regulate Golgi stability during mitosis (53). High *HOOK3* expression could predict a poor prognosis for prostate cancer (54). Future research should focus on elucidating the underlying molecular mechanisms and conducting functional studies to validate the clinical significance of such cis-regulatory editing.

Functional enrichment analysis showed that apoptosis and necroptosis mainly enriched PCOS-associated RNA editing. In previous studies, apoptosis and necroptosis have been reported to be linked with PCOS. For instance, SH2B adaptor protein 3 (SH2B3, also called LNK) could promote GC apoptosis through the AKT-FOXO3 pathway (55). Apoptosis could also be regulated by the toll-like receptor TLR8 (56). In addition, apoptosis and necroptosis were involved in the modulation of hyperandrogenism (57).

Our findings also reveal decreased *ADARB1* expression in PCOS correlated with seven downregulated DRE sites. In ovarian cancer, the downregulation of *ADARB1* has been reported to have a potential role in the development of ovarian cancer (58). These edited genes could exert a related role in various diseases. For instance, *CCDC69* has been associated with immune infiltration and serves as a prognostic marker in breast and colon cancers (59, 60). Polymorphisms in *SLC47A1* are associated with type 2 diabetes (61, 62). Additionally, the hyperediting of *FAM126B*, represented by *FAM126B*:chr2:200978748 and *FAM126B*:chr2:200978747, might potentially contribute to PCOS. Taken together, such findings indicated a substantial role of *ADARB1* and RNA editing mediated by it in the pathophysiological process of PCOS.

RBPs play a crucial role in regulating post-transcriptional processes, including RNA splicing, decay, and editing (63, 64). Importantly, our findings suggest that RBM45 is the RBP with the highest number of binding sites in the identified differentially edited genes. Previous studies have reported the regulation of splicing machinery by RBM45 in liver biopsies from patients with nonalcoholic fatty liver disease (65). Furthermore, RBM45 has been shown to bind to N6-methyladenosine, thereby controlling and regulating mRNA processing (66). These results highlight the possible regulatory role of RBPs in the biological functions of RNA editing in the context of PCOS.

In conclusion, our study conducted a transcriptome-wide analysis of PCOS-associated A-to-I RNA editing sites and provided new insight into understanding the role of RNA editing in the pathogenesis of PCOS.

## Data availability statement

The original contributions presented in the study are included in the article/**Supplementary Material**. Further inquiries can be directed to the corresponding author.

## Author contributions

FSK and ZJL contributed equally to this article. FSK contributed to drafting the manuscript. YPM, ZJL and JHW revised and improved the writing of the manuscript. YZ performed the data analysis. YHL, RFJ, BLZ, PWH and CYR contributed to the revision of the manuscript. JHC and YPM designed the study and revised the manuscript. All authors contributed to the article and approved the submitted version.

## Supplementary material

Supplementary Figure 1 The single-nucleotide variant density in various chromosomes.

Supplementary Figure 2 The sequence preference of the A-to-I RNA editing sites in PCOS.

Supplementary Figure 3 Principal Component Analysis of the samples of PCOS and control.

Supplementary Figure 4 The editing level of Cis-Regulatory editing sites.

Supplementary Figure 5 The word cloud of the types of RBPs targeting differential editing sites.
