# Supplementary figures and images for: Transcriptome analysis identification of A-to-I RNA editing in granulosa cells associated with PCOS
Source: Front Endocrinol (Lausanne). 2023 Jul 21;14:1170957. doi: 10.3389/fendo.2023.1170957 (PMC10401594; doi:10.3389/fendo.2023.1170957)

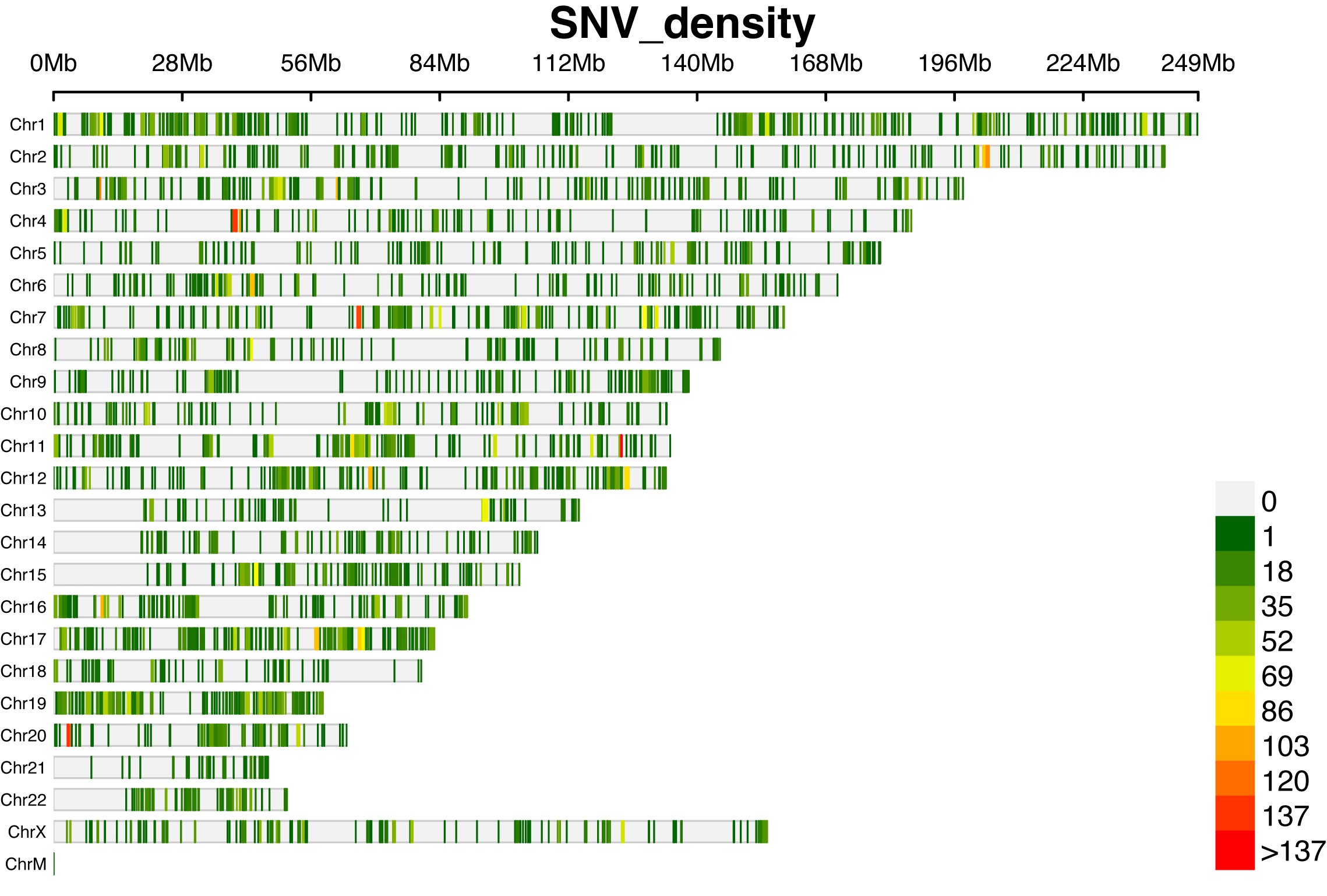

Supplement: Supplementary Figure 1 — The single-nucleotide variant density in various chromosomes. [file Image_1.tif]

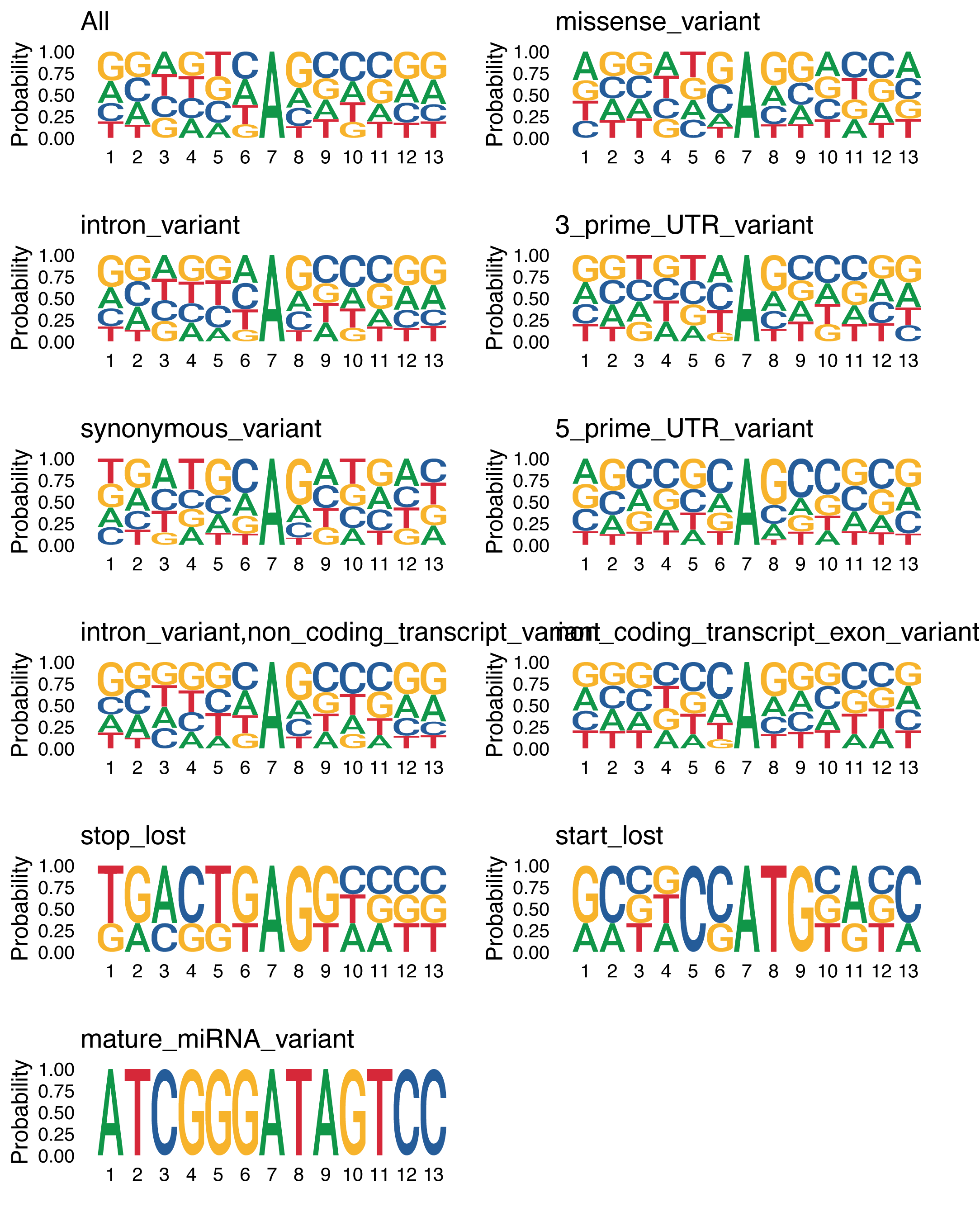

Supplement: Supplementary Figure 2 — The sequence preference of the A-to-I RNA editing sites in PCOS. [file Image_2.tif]

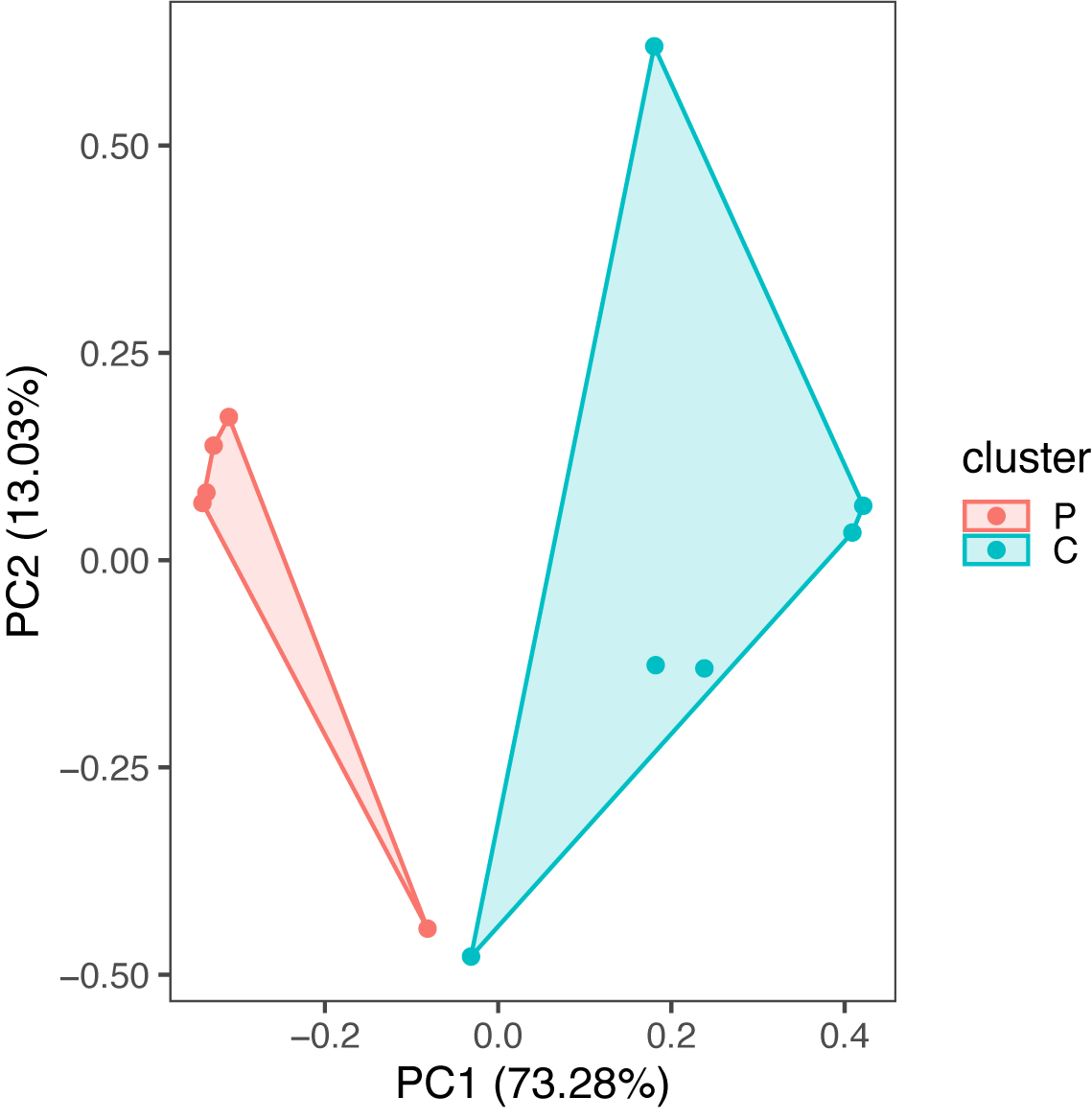

Supplement: Supplementary Figure 3 — Principal Component Analysis of the samples of PCOS and control. [file Image_3.tif]

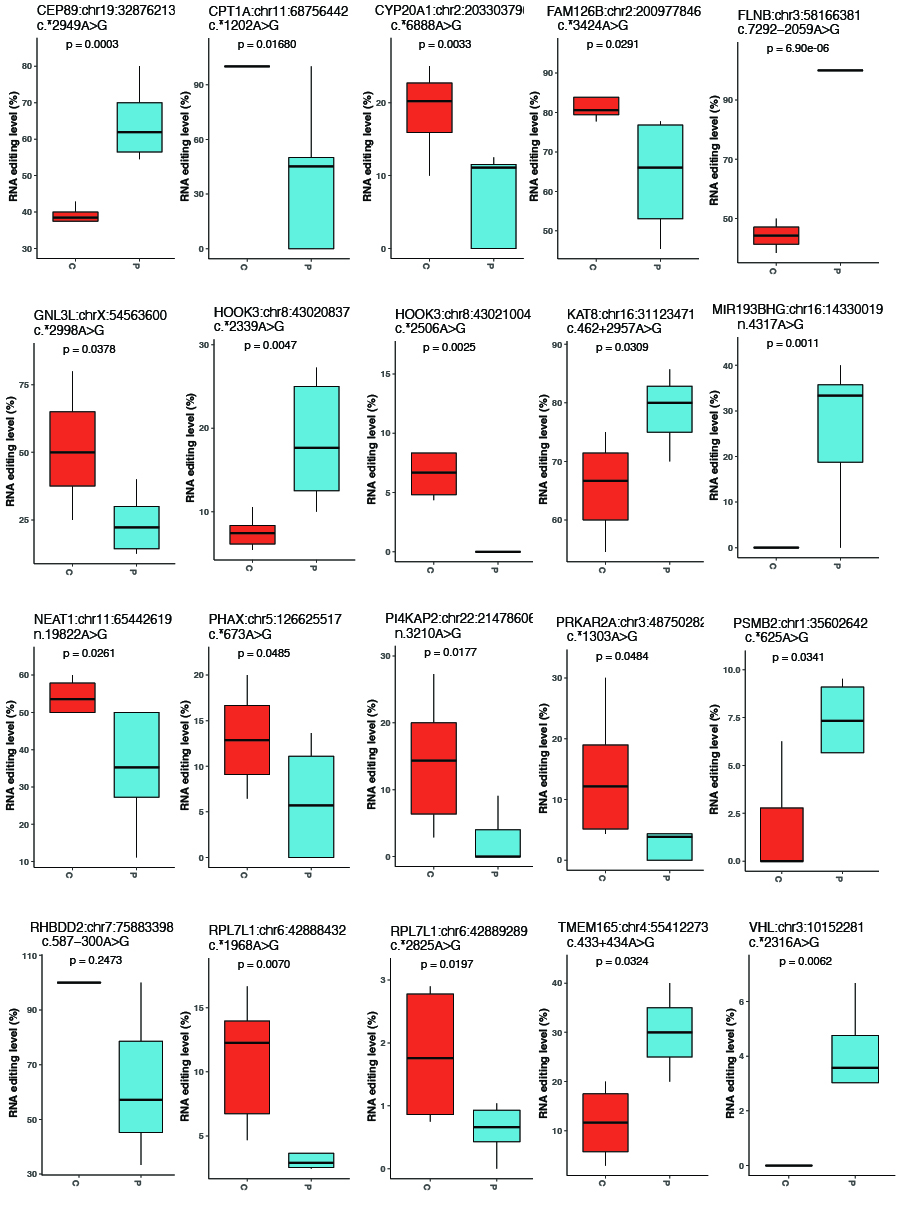

Supplement: Supplementary Figure 4 — The editing level of Cis-Regulatory editing sites. [file Image_4.jpeg]

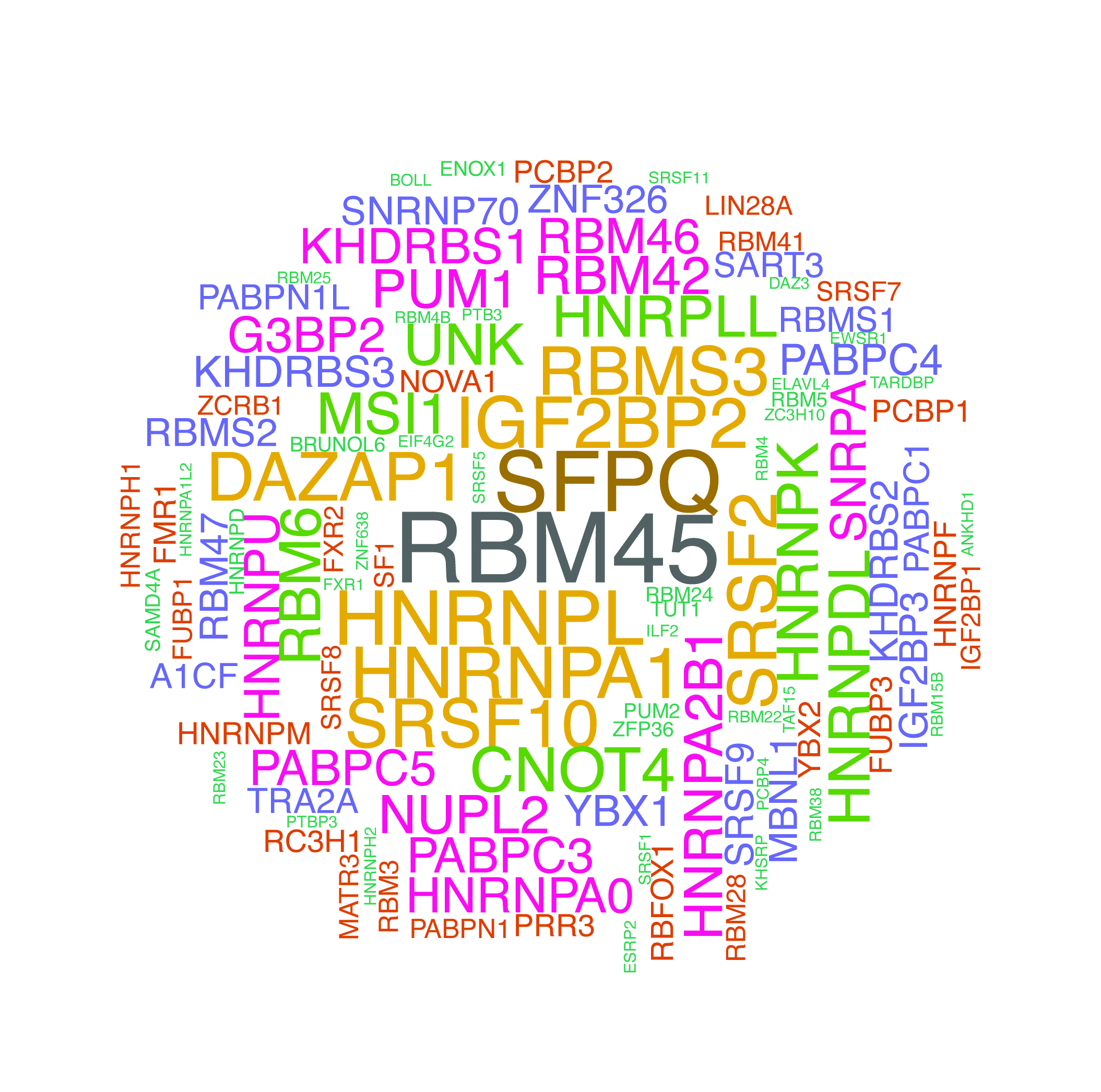

Supplement: Supplementary Figure 5 — The word cloud of the types of RBPs targeting differential editing sites. [file Image_5.tif]
